# Intra-Uterine Variola

**Published:** 1866-04

**Authors:** 


" *Intra-Uterine Variola.*—M. Legros presented to the Biological Society of Paris a variolous fœtus, with the following history:—On May 18th, a woman in the Hotel Dieu was prematurely delivered of a fœtus aged apparently about five months, which was covered with pustules or small-pox. The mother had distinct marks of vaccination, and had never had small-pox. About six months previously, she had had connection with a man who was convalescent from variola. No exposure of the mother to contagion could be traced. This case, Mr. Legros observed, raised the question whether the father could have communicated the small-pox at the moment of fecundation, the disease remaining for five months in a state of latency. This theory he believes to be supported by the facts that, when a pregnant woman has small-pox, the fœtus is sometimes not attacked till some time after the recovery of the mother; and that, in a child of a syphilitic father, the disease in some cases does not show itself in the infant until several days or even weeks after birth."

The above case is copied from the Buffalo *Medical and Surgical Journal*, as taken from *Gazette Medical de Paris*, August 5, 1865.

A case, in some respects similar to this, was reported to the Atlanta Medical Society, in February last, by Dr. H. L. Wilson. He stated that a few days before, he was called to see a lady in labor, and soon after his arrival she was delivered of a dead fœtus, apparently about the eighth month, which was covered with the characteristic small-pox pustule. At the time of delivery he was at a loss to account for the disease in the fœtus, as the mother disclaimed having had any variolus affection. At a subsequent visit, however, a more thorough investigation convinced him that the mother had had a light attack of varioloid a few weeks previous to her premature delivery.

Is it not probable that in M. Legros' case a light attack of varioloid had passed unnoticed by the mother?

